# A scalable and reliable deep learning framework for enhanced brain tumor detection and diagnosis using AI-based medical imaging

**DOI:** 10.3389/fmed.2026.1738796

**Published:** 2026-01-30

**Authors:** Sultan Ahmad, Stephen Neal Joshua Eali, Thirupathi Rao Nakka, Naceur Chihaoui, Tahani Alsubait, Humaira Khanam

**Affiliations:** 1 Department of Computer Science, College of Computer Engineering and Sciences, Prince Sattam Bin Abdulaziz University, Alkharj, Saudi Arabia; 2 School of Computer Science and Engineering, Lovely Professional University, Phagwara, Punjab, India; 3 Department of Computer Science and Engineering, Gandhi Institute of Technology and Management GITAM University, Visakhapatnam, Andhra Pradesh, India; 4 Department of Computer Science and Engineering, Vignan’s Institute of Information Technology (A), Visakhapatnam, Andhra Pradesh, India; 5 Physics Department, Preparatory Year Deanship, Prince Sattam Bin Abdulaziz University, Al-Kharj, Saudi Arabia; 6 Department of Computer Science and Artificial Intelligence, College of Computing, Umm Al-Qura University, Makkah, Saudi Arabia; 7 Department of Health and Rehabilitation Sciences, College of Applied Medical Sciences, Prince Sattam Bin Abdulaziz University, Al-Kharj, Saudi Arabia

**Keywords:** BraTS-2015, classification, CNN, computer aided diagnosis, deep learning, DeepLabV3, inception net V3, medical imaging

## Abstract

**Background:**

The proposed Architecture will provide the processing and analysis essential to accurate and reliable detection of brain tumors from MRI, for timely diagnosis and evidence-based decisions. Medical imaging now routinely enters clinical assessment; the thrust is shifting toward attaining high performance within open and governed systems to enable deployment in real-world healthcare applications.

**Methods:**

This paper proposes a two-stage deep learning framework: first, DeepLabV3 for segmentation to demarcate candidate tumor regions, and then CNN to classify whether a tumor exists. The different components employ pre-trained models through transfer learning and fine-tuning. The DeepLabV3 and CNN architectures are used, together with metric computation modules. This approach will be tested on BraTS MRI data. For efficient model training, optimizers such as SGD, RMSprop, and Adam can be employed.

**Results:**

The classification performance could be achieved with a high value of 99.31% using an ADAM optimizer in the proposed architecture. Besides, both the precision and recall are very high, indicating good generalization and stable performance. Moreover, segmenting before classification provides more reliable detection compared to using a single-stage model.

**Discussion:**

These results indicate that feature learning guided by segmentation enhances tumor detection with a binary classifier, while remaining interpretable and robust. This makes the framework much more transparent and easy to audit, suitable for use in cloud-enabled, secure, and IoT-enabled clinical environments.

**Originality:**

It therefore proposes a two-layer deep learning architecture that effectively incorporates precise tumor localization into explicit binary tumor detection. Beyond this, the work focuses on practical clinical applicability, robust data governance, and deployment-ready systems rather than diagnosing subtypes of tumors.

## 1 Introduction

The Brain roughly 1 billion neurons (1, 2) fire off electrical and chemical signals (3, 4) that define our reality. The brain controls perception, mood, and personality, while the cerebellum helps maintain balance and coordinates movements with the cerebral cortex (4). All brain systems interact to enable everyday behavior, but they can be impaired by disease. A tumor refers to an abnormal growth of cells that forms a lump (5). Tumors can be benign, slow-growing, or malignant, fast-growing (6, 7) and able to invade surrounding tissue. They can be intrinsic, arising within the brain, or extrinsic, arising elsewhere and extending into the brain.

Clinicians use imaging and biopsies (8) to further describe the disease and its extent of spread. Origin-based classifications have informed prognosis, treatment selection, and challenges in diagnosis. Diagnostics and therapeutics (9) in neuro-oncology continue to evolve, with much work still to be done.

Models trained for different tasks commonly reuse representations learned on vast medical images, with good performance using limited data. The general models include VGG, ResNet, Inception, MobileNet, and DenseNet. The detection of patterns in medical images is influenced by model depth and complexity (10). Cancer detection and classification will be greatly improved by the rapid development of leveraging pre-trained models to contribute toward personalized care.

With deep learning, AI, and transfer learning, significant enhancements are possible. A balance between feature extraction and computational efficiency is achieved with a fine-tuned CNN, DeepLabV3, which handles the processing of medical images to support rapid and accurate disease classification, characterization, and diagnosis (11, 12). Applications include brain, lung, and breast cancers, all diagnoseable with extremely high accuracy. Higher resolution might be achieved with a bilayer configuration. Clearly defined brain regions and lesions also enable surgical planning and improve quality of life. This technique opens new frontiers in personalized screening and follow-up, allowing prediction of problems and recurrences and providing information on the treatment effectiveness. This paper proposes a bilayer deep learning framework that automatically detects brain tumors from MRI scans. This approach couples the image segmentation module (DeepLabV3) to precisely locate the candidate regions of a tumor and CNN as a classifier to classify MRI scans into either a tumor or a non-tumor case. To further enhance its robustness, the paper has incorporated transfer learning with fine-tuning to boost feature representation. In this research, MRIs showing gliomas, meningiomas, or pituitary tumors are combined to form the single class of tumor and frame the problem in terms of binary decisions: tumor versus non-tumor. Our work is intended for early-stage screening to provide computationally efficient and clinically interpretable decision-support rather than subtype-specific tumor diagnoses. It offers improved performance in terms of detection and classification compared to previously available techniques, along with enhanced segmentation accuracy.

## 2 Literature review

It is not common for researchers to integrate brain cancer identification with DeepLabV3, InceptionV3, and CNN for classification. More precise classification and segmentation must be carried out to enhance brain tumor identification from the chosen dataset.

Some of the earlier works that presented the tumor identification with different types of datasets and their performances are discussed. In a work (13), the authors used the basic CNN model to identify and classify the brain tumor from the BraTS-2013 dataset and secured an accuracy of around 79.8%. In another work (14), the authors had combined the CNN model with other ConvNets for the same brain tumor identification by using the BraTS-2013 dataset and secured an accuracy of 94% in identifying or classifying the brain tumor in the human brain MRI MRIs. In Banerjee et al. and Cham et al. (15, 16), the authors used the SVM algorithm in classifying the brain tumors from the dataset chosen by the authors, which is from the RIDER dataset. The authors had achieved an accuracy of 93.1% in classifying the affected scanned MRIs from the total set of scanned MRIs. The authors used the NNU-Net model to classify the presence of brain tumors on the selected input data given to the model (17). To verify the performance of the proposed model, the authors tested the model on the dataset, namely BraTS-2015, for the identification of brain tumor. By using the same method, the authors had achieved an accuracy of around 88.9% with very high execution time.

In a work (18), the authors proposed a hybrid model that combines a CNN model with an SVM algorithm for the classification of brain tumors from the selected dataset of scanned MRIs. In Xun et al. (19), the authors proposed a hybrid model that combines both ResNet and InceptionV2 for the same detection of brain tumors from scanned input MRIs. By observing all these existing models, the individual model’s performance is very less when compared with the performance of a hybrid model in the identification of tumors. Hence, a hybrid model is required to achieve better accuracy in the identification of brain tumors and to achieve good output at a faster interval of execution time. To achieve these problems, we had proposed a propose a Bilayer architecture model to enhance brain tumor diagnosis. It comprises of combining the DeepLabV3 for segmenting input images and the other InceptionV3 model for feature extraction from the segmented images. The Convolutional Neural Network (CNN) is the next layer of the proposed model for classification of brain tumor images. A schematic diagram displays our suggested Bilayer architecture for the detection of brain tumors.

To improve understanding of earlier work related to the proposed model and its performance against different datasets and performance measures, some earlier works are illustrated in Table 1 in tabular format. They include the employed classifiers, information pertaining to the datasets, and accuracy obtained by the earlier models.

**Table 1:** Comparison of various existing models.

Ref	Classifier	Dataset	Data governance compliance	XAI support	Clinical readiness level (CRL)	Limitations	Deployment environment	Output
(13)	CNN design	BRATS2013	Not Addressed	None	CRL-1	Shallow Model	Offline research	79.8%
(14)	VGG + ConvNets	BRATS2013	Partial	None	CRL-2	High Computational Cost	Local Workstation	94%
(15)	SVM	Harvard, RIDER	Not Addressed	None	CRL-2	Manual Feature Extraction	Offline research	93.1%
(16, 17)	NNU-Net	BRATS2013	Partial	None	CRL-2	High inference Time	Research Server	88.9%
(18)	SVM + CNN	BRATS2013	Partial	None	CRL-1	Limited Generalization	Local Workstation	96.4%
(19)	RGA-Unet	TCIA	Partial	None	CRL-2	Complex Architecture	Cloud Based	95.04%
(20)	InceptionRes NetV2	BRATS2020	Partial	None	CRL-2	Large Model Size	Offline research	96%
(33)	VGG-16	BRATS2020	Not Addressed	None	CRL-2	Overfitting Risk	Research Server	88.9%
(34)	Unet++	Hybrid	Partial	None	CRL-2	Limited robustness	High End Workstation	89.25%
(35)	3D Unet	BRATS2020	Partial	None	CRL-2	High Memory Usage	Research Server	86%
(36)	Segmentation Network U-Net	TCIA	Partial	None	CRL-1	Large Model Size	Offline research	94.57%
(21)	ResNet50-Unet	CVC Clinic DB	Partial	None	CRL-2	Limited Generalization	Offline research	91.2%
(22)	CNN Based Inception V3	TCIA	Partial	None	CRL-2	Large Model Size	High End Workstation	95%
(23)	Hyper Parameter Tuned CNN	MRI	Full (Privacy, auditability, interpretability)	Model-Level Transparency	CRL-3	Mixed	Cloud & IoT enable Clinical System	99.31%

## 3 Methodology

This section provides an explanation of the materials and procedures employed in the model.

### 3.1 Dataset overview

To ensure validation of the proposed method, we employed the BraTS-2015 dataset with 274 MRI brain tumor scans (20, 21). Images of the ‘no tumor’ class were also included for comprehensive coverage of different brain pathologies. Each image is annotated for proper training and evaluation of the segmentation algorithm. Data augmentation boosted the dataset to approximately 765 brain scans. BraTS-2015 has an important role in the accurate location and delineation of brain tumors, which is a basic requisite for diagnosis and treatment planning. The dataset contains images with and without tumors to allow for robust segmentation. A large collection of various tumor types, each with anatomic variation, improves the performance and increases the clinical value of the model. The model was tested using 755 augmented images (22).

### 3.2 Pre-processing pipeline

The proposed model requires MRI data to undergo preprocessing before any further analysis. Resizing the image is performed to achieve a standard dimension; hence, the size of the input is maintained. Normalization of contrast changes the intensity levels so that salient features are more detectable by the model (23, 24). This includes the addition of image augmentation techniques like rotation, flipping, and scaling to increase the dataset in order to expose the model to different conditions. Skull stripping in MRI processing removes non-brain tissues while maintaining focus on the brain structure for more accurate tumor detection. The pipeline for preprocessing maps the original MRI image to the predicted final mask. The outline of the tumor region is delineated in red for easy identification (25, 26).

### 3.3 Augmentation strategy

Data augmentation includes methods that artificially increase the size of a training dataset in order to improve deep learning model performance. New records of data are created from existing ones by involving intentional changes or known procedures; this is included in references (23, 27). Accuracy in most machine learning algorithms generally increases with diversified samples. Hence, using varied datasets leads to better performance and a more accurate model.

### 3.4 DeepLab V3

**Figure 1:**
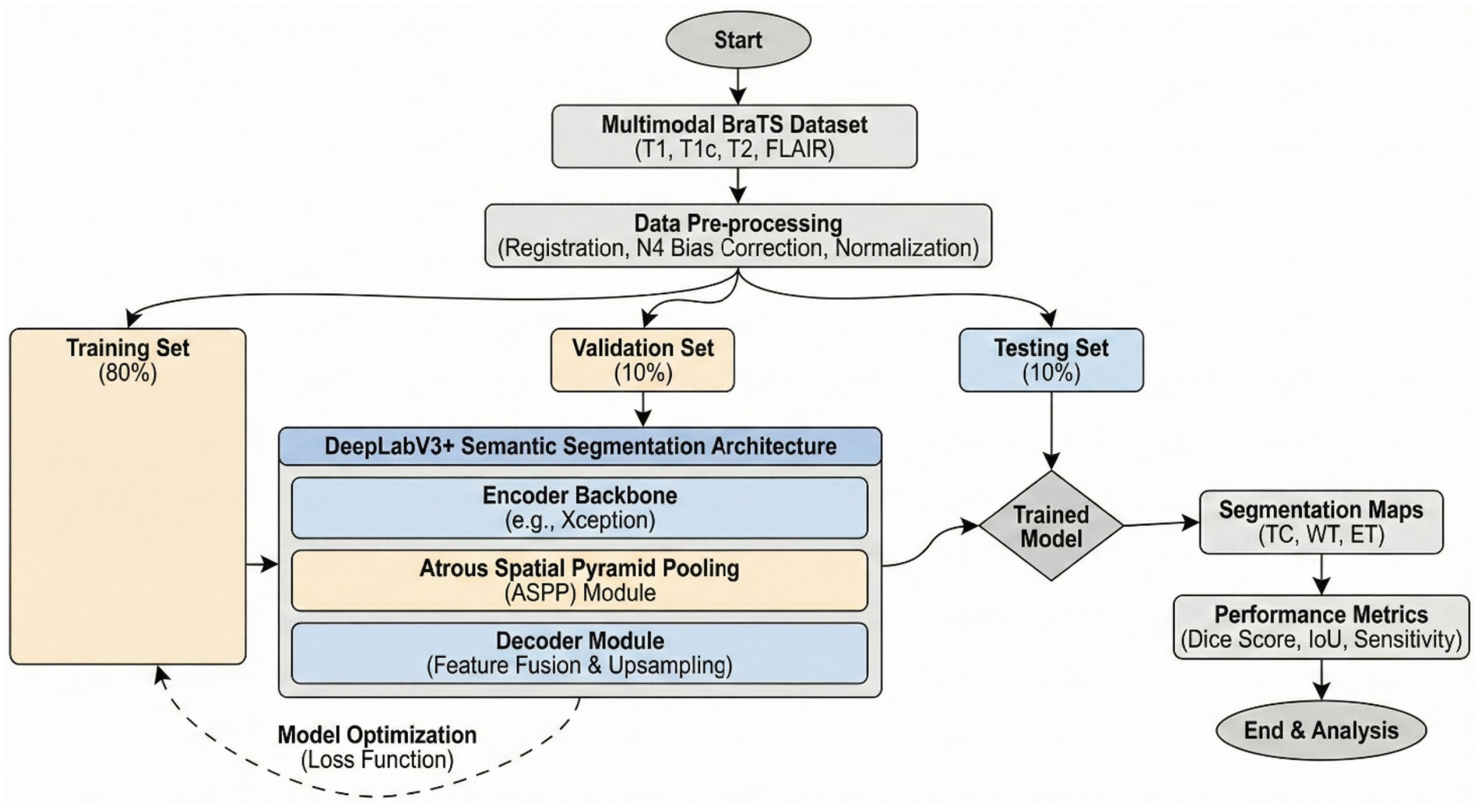
Flowchart of DeepLabV3.

The encoder module uses dilated convolution to capture contextual information at multiple scales, while the decoder iteratively refines object boundary segmentation. The DeepLabV3 is adopted in this network as an encoder module and further enhances edge-information extraction. The decoder reconstructs features for the predicted outputs and thus improves the effectiveness of segmentation with a better preservation of edge information. The Xception network is used by DeepLabV3 as the backbone. It also employs deep separable convolution between the ASPP and decoder modules. Therefore, improving the architecture further enhances segmentation performance. The architecture of the DeepLabv4 model in Figure 1 is visualized.

### 3.5 Inception V3

InceptionV3 uses an efficient CNN architecture which presents a good trade-off between computational speed and classification accuracy. Convolutions are factored: large kernels are shrunk to smaller, repeated ones, for example, replacing a single 5 × 5 kernel with two stacked 3 × 3 kernels. This reduces the total number of parameters and computation required while preserving the same receptive field size. Asymmetric convolutions are also used to further reduce computational expenses without reducing the ability to differentiate effectively between spatial patterns. These techniques allow InceptionV3 to be deeper while being computationally efficient compared to its previous architectures. An auxiliary classifier is strategically placed in the network’s intermediate layer to ease the flow of gradients during training and hence improve the stability of the learning process. This component is beneficial for model convergence during training. The combination of factorized convolutions, efficient layer construction, and use of an auxiliary classifier all contribute to making InceptionV3 an efficient model of image classification and feature learning.

## 4 Bilayer architecture

**Figure 2:**
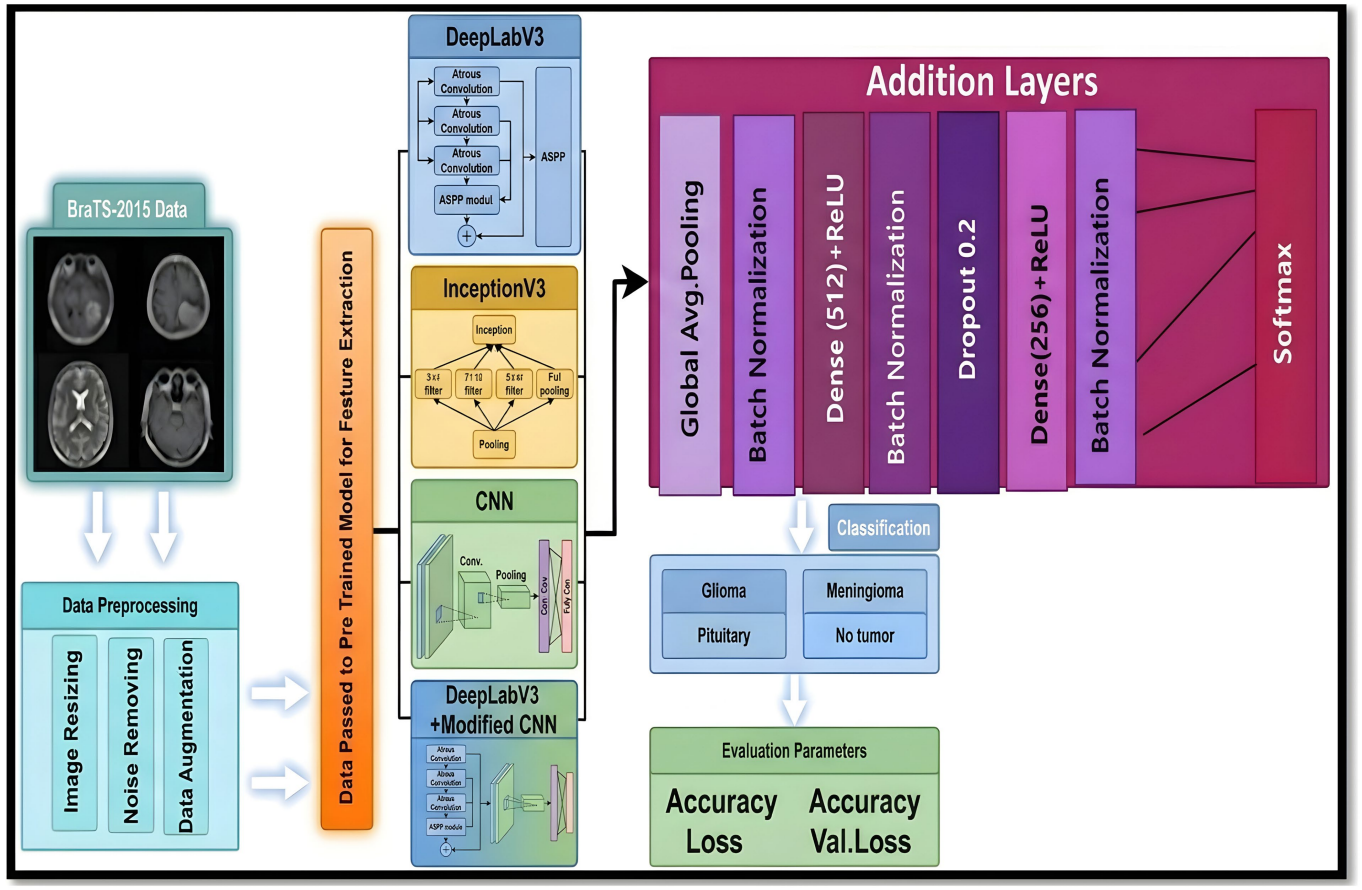
Schematic diagram of the bilayer structure.

Figure 2 illustrates the general graphical model representing the schematic diagram specifically related to the bilayer framework for the detection of brain tumors. The diagram gives an overview of the developed bilayer deep learning model designed for brain tumor classification. The entire process starts with some fundamental data preprocessing steps, which include resizing the images such that their dimensions become standard, elimination of noise for better enhancement of image quality, data augmentation methods to add diversity in the training set. After these crucial preprocessing steps are completed, the preprocessed input data are passed through a sequence of pre-trained models, which include DeepLabV3, InceptionV3, CNN, and a specially designed DeepLabV3-CNN for the purpose of deep feature extraction. The feature extraction process is followed by passing the pre-extracted features through a complex sequence of additional layers. These layers include global average pooling to reduce information, batch normalization to stabilize the learning process, dense layers using ReLU activation functions, for example, to add non-linearity, and dropout layers for preventing overfitting. Finally, the final classification process is realized by using a SoftMax layer, which discriminates effectively between the different types of tumors, i.e., glioma, meningioma, pituitary tumor, and cases where no tumor exists. To determine the performance and effectiveness of the model, evaluation is performed using significant measures, which include accuracy, loss, and validation loss, thus giving a complete understanding of its capability.

## 5 Model variant evaluation

The proposed model utilizes DeepLabV3 for segmenting the input images and a CNN with adjustments for final classification, which was accurate in terms of efficiency and stability of brain tumor segmentation and classification. In this paper, we discuss a detailed description of the proposed scheme of brain tumor feature extraction, segmentation, and classification. The proposed study adopts a systematic approach in order to attain maximum accuracy and reliability of brain tumor imaging analysis using the BraTS-2015 dataset (28–31). Initially, the dataset is divided into three equal sets: training, validation, and testing. The proposed model was trained in a robust manner, utilizing a data augmentation technique to attain maximum diversity of the training dataset. Random rotation, horizontal, and vertical flip, and random change in brightness were utilized to attain maximum robustness of the model against over-fitting. The dataset was divided into 80% training, 10% validation, and 10% testing sets to ensure the model was adequately trained and tested on unseen data. Training used a cross-entropy loss function specifically designed for multi-class segmentation tasks. Early stopping was utilized to avoid overfitting, tracking the validation loss during training (32).

### 5.1 Model variant implementation and setup

The specification units employed in every experiment are as follows: a module of 16 GB RAM, an Intel(R) Core (TM) i7-7700 Central Processor with a frequency of 2.80 GHz, and NVIDIA GTX 1050 Ti graphics cards employed in experiments. It takes around 01 h, 12 min, and 48 s to train the model and perform segmentation testing on the dataset. The programming language employed in this study is Python, and specifically, the Image Data Generator module of the Keras library is employed in order to develop sets of magnetic resonance imaging (MRI) images. Data preparation is done in real time.

### 5.2 Training methodology

This model was trained with data augmentation methods to promote diversity in the training set. To improve overfitting resistance, random rotation, flipping, and brightness modifications were used. The data set was split with a ratio of 80% training, 10% validation, and 10% testing, allowing for accurate measurements on new, unseen data. A cross-entropy loss function created especially for multi-class segmentation problems was utilized during training. Early stopping was also included to check validation loss and avoid overfitting.

### 5.3 Class imbalance mitigation strategy

All the performance differences of the proposed bilayer model with that of the baseline models were assessed for all experiments using the nonparametric Wilcoxon signed rank test, with significance defined at *p* < 0.01. This verification proved that the observed improvement was statistically significant. Class imbalance in the proposed framework was dealt with by incorporating weighted categorical cross-entropy loss along with oversampling of minority tumor subtypes.

### 5.4 Confusion matrix description

**Figure 3:**
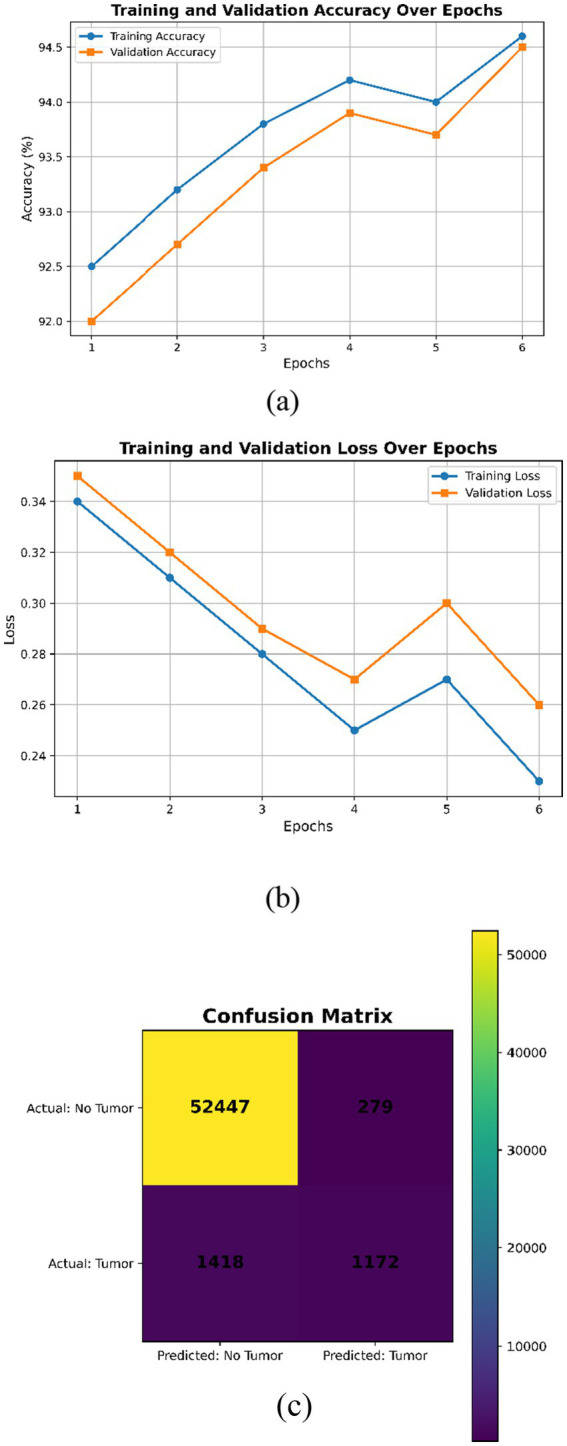
DeepLabV3 **(a)** Training and validation accuracy over the epochs; **(b)** training and validation loss over epochs; **(c)** confusion matrix.

**Figure 4:**
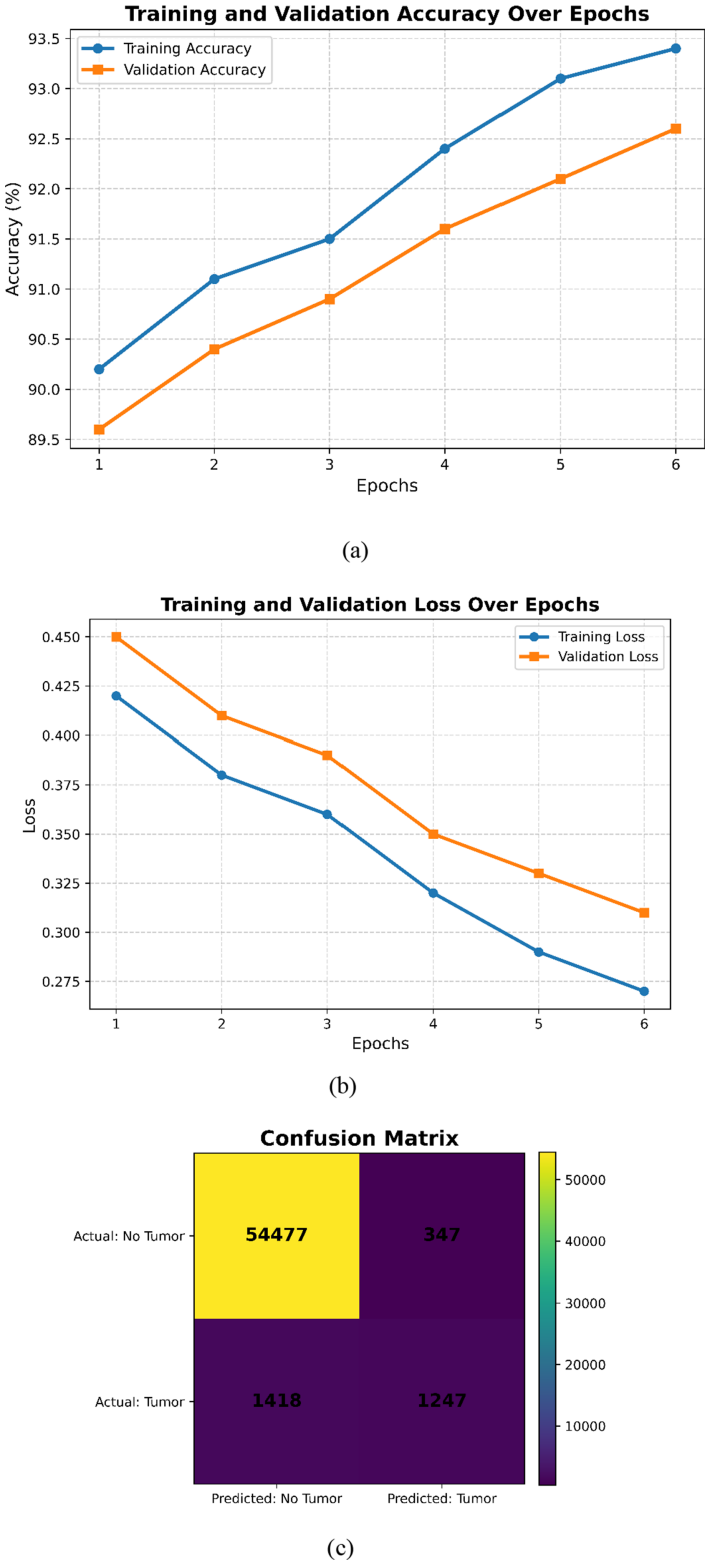
Inception V3 **(a)** Training and validation accuracy over the epochs, **(b)** training and validation loss over epochs, **(c)** confusion matrix.

**Figure 5:**
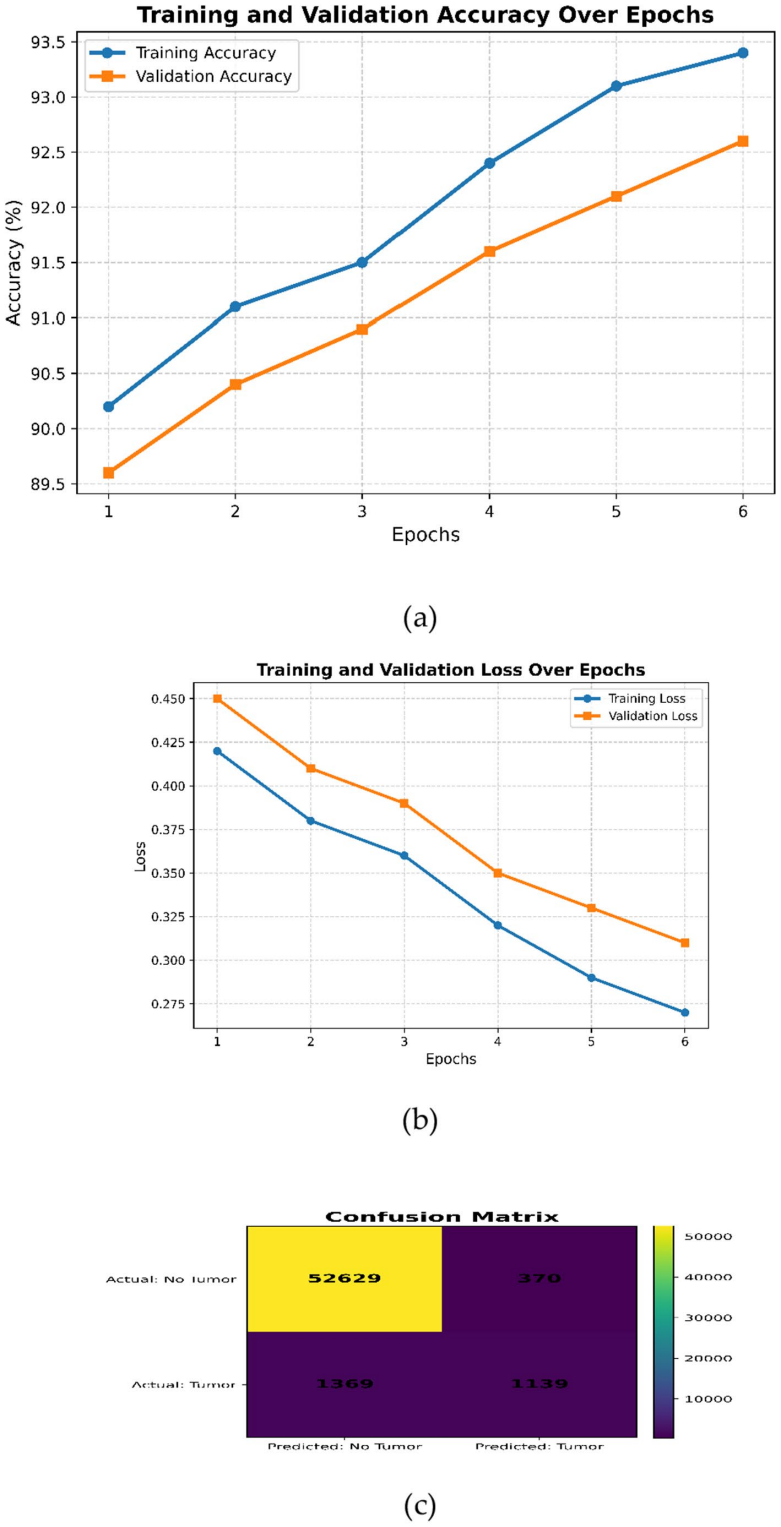
Modified CNN **(a)** Training and validation accuracy over the epochs, **(b)** training and validation loss over epochs, **(c)** confusion matrix.

**Figure 6:**
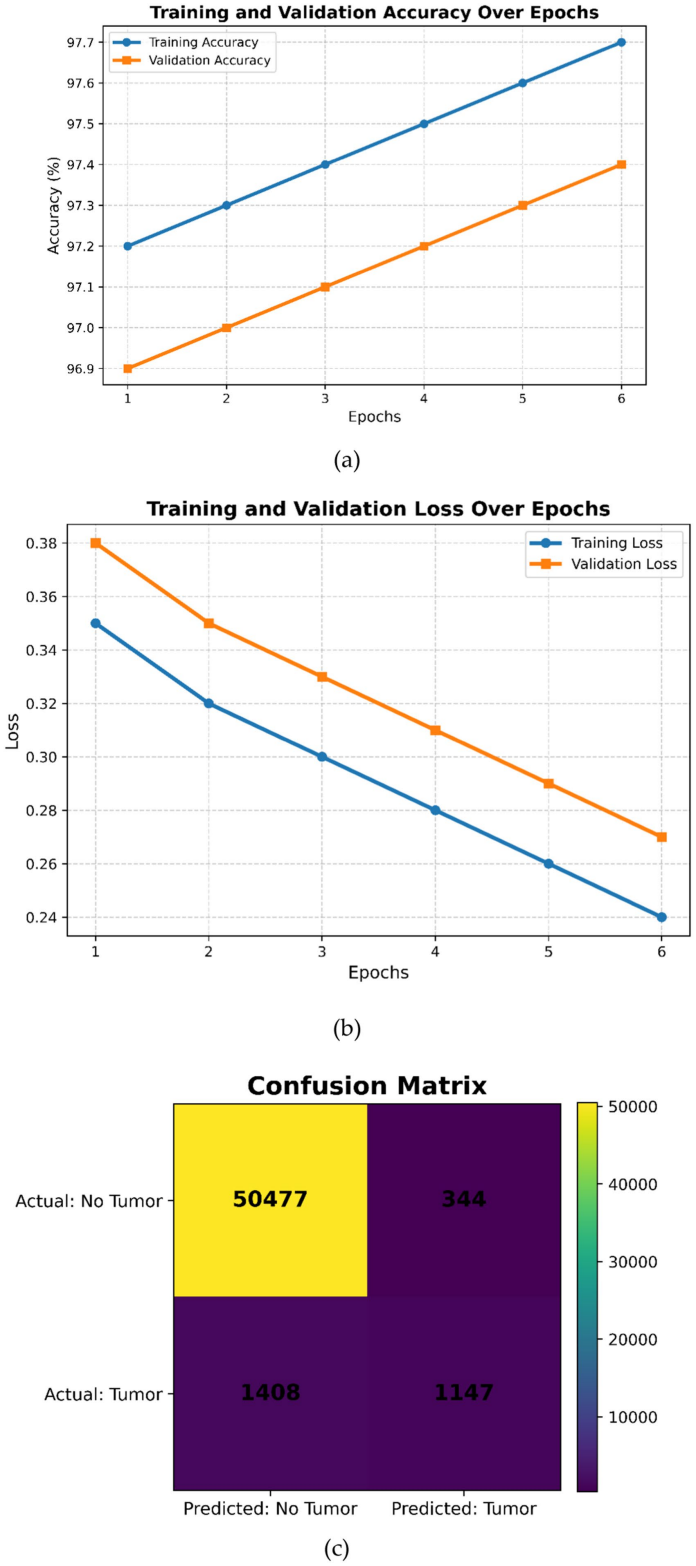
**(a)** Training and validation accuracy over epochs, **(b)** training and validation loss over epochs, **(c)** confusion matrix.

This expanded dataset consists of 755 images, of which 75 are reserved for testing according to an 80–10–10 split. For Figures 3–6 in the current work, the confusion matrices show numerical values clearly labeled as TP, TN, FP, and FN, from which the corresponding accuracy, precision, recall, and F1-score can be identified directly by the number count of samples within the test set.

## 6 Model implementation

The proposed CNN architecture for tumor classification begins with an input layer of dimension (224, 224, 3) to accommodate RGB images. It is followed by a series of convolutional layers, which start with 32 filters of size (3 × 3) and expand further to 128 filters. A pooling layer is introduced after every alternative convolutional layer to reduce the spatial dimensionality. The higher-order features are successively extracted through these layers. Further, the feature maps are flattened before being fed into a fully connected dense layer with 1,024 units to calculate the extracted features. An optional dropout layer can also be used to mitigate overfitting. The output layer comprises a single unit with a sigmoid activation function to support binary classification. Additional convolutional layers are added to improve the performance of classification, and dropout regularization has been used to improve the model’s ability to understand complex patterns in data. Several iterations during training using different pre-trained models and other variants of optimization algorithms, such as SGD, ADAM, and RMSprop, are performed for better performance in tumor classification.

### 6.1 Case 1: DeepLabV3

Generally speaking, pixel-wise classification means the capability for every pixel of an image to be assigned as a label by the DeepLabV3 model. There are two forms of semantic segmentation supported by the model: one is for object delineation through boundary delineation. DeepLabV3 is applied to tumor segmentation, locating tumors amidst surrounding tissues by extracting boundary information. Faster convergence during the training process benefits from transfer learning, which leverages pre-trained DeepLabV3 weights. Case 1 reaches an accuracy of 94.80%, indicating that the performance in tumor segmentation will be improved. The corresponding flow chart is given in Figure 1.

The peak performance is realized at Epoch 6, which is characterized by the following values for the optimal metrics: Accuracy = 94.80%, Recall = 0.92, Precision = 0.94, Sensitivity = 0.93 and F1 Score = 0.95. The reduced standard deviations are a testimony to the model’s predictability. Table 2 shows the increased stability of the DeepLabV3 model with an increased number of training epochs.

**Table 2:** Evaluation metrics of case 1.

Epoch	Acc (%)	Recall	Precision	Sen	F1-Score	SD
1	93.60	0.90	0.90	0.92	0.91	0.03
2	93.80	0.90	0.92	0.92	0.92	0.03
3	93.96	0.91	0.93	0.92	0.92	0.02
4	94.20	0.91	0.93	0.93	0.93	0.02
5	94.60	0.92	0.94	0.93	0.94	0.01
6	94.80	0.92	0.94	0.93	0.95	0.01

The training and validation curves in Figure 3a show gradual improvements; hence, the model learns well without overfitting. Figure 3b shows progressive smooth decrease of both training and validation loss, indicating good convergence without under- or overfitting. Overall, Figure 3 indicates a well-trained DeepLabV3 model with high accuracy and low loss, although there is a small gap between training and validation metrics, indicating good generalization. Figure 3c shows high performance on negative classifications but relatively poorer performance on positive classifications. It should be possible to improve recall without affecting precision by enhancing the learning of positive examples through class balance or alternative hyperparameter searching.

### 6.2 Case 2: InceptionV3

**Figure 7:**
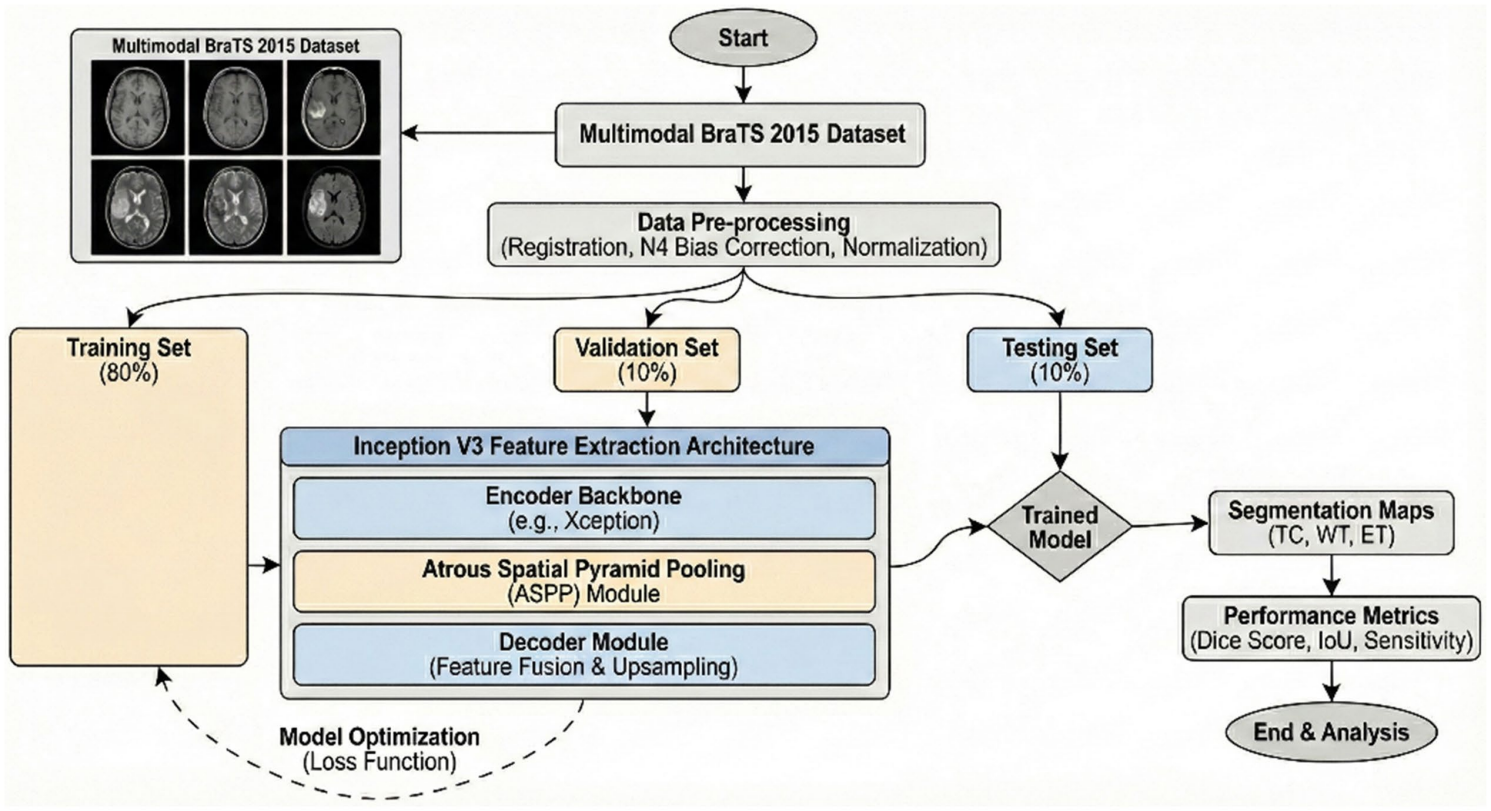
Flow chart of inception V3 model.

These features are derived from MRI images that include information about structure, texture, and abnormalities. For neoplasm features, the InceptionV3 architecture is used, extracting features of multiple scales using its deeper layers. This work uses transfer learning, wherein the pre-trained weights of InceptionV3 enhance training performance and augment its efficiency. Case 2 illustrates tumor extraction, which yields an accuracy of 91.89%. Figure 7 presents the flow diagram for Case 2.

Model reaches the optimal performance at Epoch 6, with metrics including: Accuracy: 92.89%, Recall: 0.90, Precision: 0.92, Sensitivity: 0.90, and F1 Score: 0.88. The standard deviation is smaller, which means that the model has more stable predictions. Table 3 shows the convergent and stable performance of the InceptionV3 model across epochs.

**Table 3:** Evaluation metrics of case 2.

Epoch	Acc (%)	Recall	Precision	Sen	F1-Score	SD
1	90.10	0.85	0.86	0.84	0.86	0.02
2	91.20	0.85	0.87	0.84	0.86	0.02
3	92.15	0.86	0.89	0.86	0.87	0.01
4	92.35	0.86	0.90	0.86	0.87	0.01
5	92.66	0.89	0.91	0.89	0.88	0.01
6	92.89	0.90	0.92	0.90	0.88	0.01

Figure 4 shows complementary observations. In subfigure (a), both curves improve monotonically, indicating good learning without noticeable overfitting issues. Subfigure (b) reflects a smooth decrease in both training and validation losses, showing good convergence without underfitting or overfitting issues. Overall, the plots reflect that InceptionV3 is well-trained, attaining higher accuracy with low loss across different epochs. The small gap between training and validation metrics is indicative of strong generalization. There is no indication of underfitting or overfitting for the model (Figure 4). The confusion matrix shown in Figure 4c reflects good performance for negative cases but relatively weaker performance for the positive ones, which may require further adjustments to handle class imbalance or tune certain hyperparameters with a view to improving recall without significant loss of precision.

### 6.3 Case 3: modified CNN

**Figure 8:**
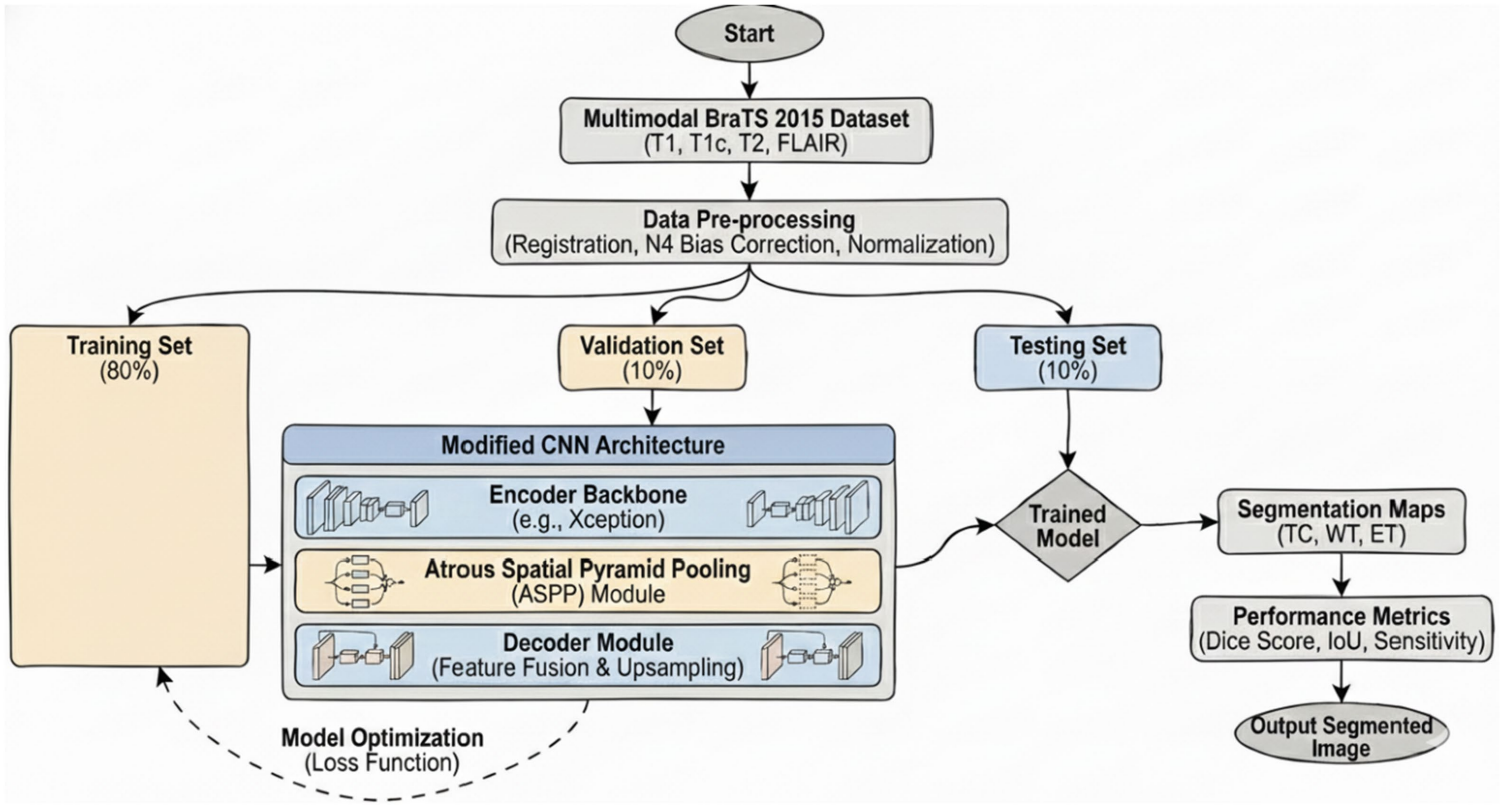
Diagram of the modified CNN.

The features encode anomaly, texture, and structural information in magnetic resonance imaging data. For the classification of tumors, a modified CNN is employed. The CNN extracts multiscale features from MRI images through its deep convolutional layers and captures the high-level structural properties of tumors effectively. Iterative model training is performed in this network through transfer learning, where pre-trained weights enable efficient training and better performance of the model. Case 3 has an accuracy of 92.72%, reflecting its robust performance in tumor extraction. The flow chart of Case 3 is given in Figure 8.

The peak performance has been at Epoch 6 with the model achieving maximum metrics of Accuracy 93.92%, Recall 0.93, Precision 0.92, Sensitivity 0.91, and F1 Score 0.91. The relatively low standard deviation suggests its predictions have been more consistent. Table 4 shows that the most recent CNN model improves in performance and consistency across epochs.

**Table 4:** Evaluation metrics of the current case 3.

Epoch	Acc (%)	Recall	Precision	Sen	F1-Score	SD
1	90.50	0.86	0.85	0.85	0.85	0.03
2	92.12	0.88	0.87	0.86	0.87	0.03
3	92.75	0.88	0.89	0.86	0.87	0.02
4	93.40	0.91	0.89	0.88	0.89	0.02
5	93.65	0.93	0.92	0.90	0.91	0.02
6	93.92	0.93	0.92	0.91	0.91	0.02

It is obvious from Figure 5a that both curves increase consistently, indicating proper learning without overfitting. Figure 5b clearly shows the convergent path for both training and validation losses, proving robust convergence without indications of underfitting or overfitting. In summary, all these plots reflect that the hyperparameters of the modified CNN model are well tuned, showing gradual increases in accuracy and decreases in loss during epochs. The metrics in training and validation show improvement continuously without any significant divergences, indicating good generalization performance and no obvious overfitting or underfitting.

The confusion matrix on test data available in Figure 5c further illustrates that the performance is much better on the negative class compared to the positive class. Recall can be enhanced by either reducing class imbalance or performing more tuning of hyperparameters toward increasing the probability of correct detection for the positive class while maintaining reasonable precision levels.

### 6.4 Case 4: DeepLabV3 with modified CNN model

**Figure 9:**
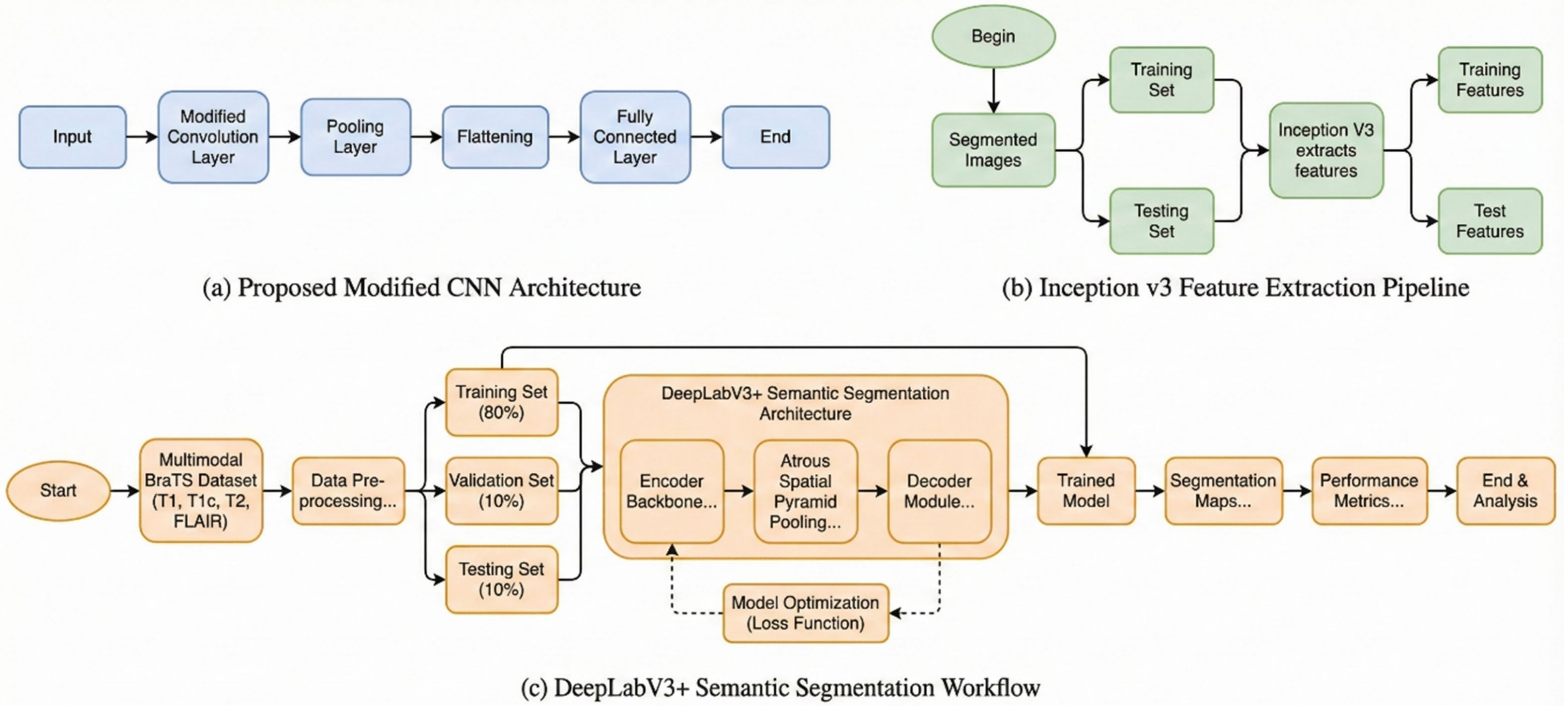
Flow chart for DeepLab V3 with modified CNN.

Case 4: DeepLabV3 is combined with a specially designed CNN in the three-layer structure for tumor detection. The tumor is segmented with high accuracy by using DeepLabV3 in the first layer to clearly demarcate the tumor from the surrounding tissues. In the third layer, the specially designed CNN is used in modified form by the addition of more convolutional and pooling layers to improve the tumor classification performance. Transfer learning of each of the three layers is used separately. This efficient integration of feature extraction, segmentation, and classification in Case 4 produced the best performance of 99.31%. Figure 9 shows the flow chart for Case 4.

It reaches the top at Epoch 6 with 99.37% accuracy, 0.97 recall, 0.97 precision, 0.97 sensitivity, and 0.97 F1 score. The standard deviations are all lower, demonstrating greater consistency of model predictions. Accordingly, the improved performance and stability of DeepLabV3, based on the enhanced CNN model, are given in Table 5.

**Table 5:** Evaluation metrics of the current case 4.

Epoch	Acc (%)	Recall	Precision	Sen	F1-Score	SD
1	97.80	0.96	0.95	0.95	0.95	0.02
2	98.20	0.96	0.96	0.96	0.96	0.02
3	98.41	0.97	0.96	0.96	0.96	0.01
4	98.62	0.97	0.97	0.96	0.97	0.01
5	98.93	0.97	0.97	0.97	0.97	0.01
6	99.37	0.97	0.97	0.97	0.97	0.01

Figure 6a shows how the two curves converge, which is indicative of successful learning without overfitting. Figure 6b shows a continual decrease in training loss and validation loss, signaling proper convergence without any overfitting or underfitting. Overall, these plots indicate that on each successive epoch, the model seems to train the DeepLabV3 + Modified CNN with an increase in accuracy and a reduction of loss. Because no divergence was seen between training and validation metrics, there is good generalization. Based on the confusion matrix, as shown in Figure 6c, it seems the model has performed very well on negative cases but not too well on positive cases. This might reveal an issue with class imbalance, or it could indicate that tuned updates are necessary to improve the performance for the positive class without compromising precision.

## 7 Experimental results and discussions

Table 6 illustrates the performance of different classification models employed for the detection of brain tumors from MRI images. The evaluation metrics are Accuracy, AUC, Sensitivity, and Precision. Conventional models, such as CNN and VGG, produce promising results with VGG yielding an accuracy of 94.01% and an AUC of 0.95. Hybrid or deep ensemble models in the form of SVM-CNN, RCNN, and InceptionV3 + V2 demonstrate further enhancements, with accuracies greater than 95% and AUC values of 0.97. In the case of a Bilayer architecture, the maximum accuracies are 98.70, 99.12, and 99.37% for Cases 1–4, respectively. The relatively small variance between these models reflects their stability. Deep CNN consistently improves the accuracy across all cases; Case 4 results in optimal performance in terms of maximum accuracy and consistency. The results are reported for single- and bi-layer configurations; each case highlights respective results.

**Table 6:** Comparative analysis of proposed model with other classifiers.

Classifier	Acc (%)	AUC	Sensitivity	Precision
CNN	79.08	0.80	0.77	0.79
VGG	94.01	0.95	0.93	0.94
SVM (RBF)	93.12	0.94	0.91	0.93
Nu-Net	88.08	0.88	0.89	0.87
SVM-CNN	96.42	0.97	0.96	0.97
RCNN	95.12	0.97	0.95	0.96
InceptionV3 + v2	96.12	0.97	0.95	0.96
VGG-16	88.97	0.88	0.87	0.89
U-Net	89.25	0.91	0.89	0.90
3D U-Net	86.36	0.88	0.85	0.86
Segmentation ResNet	94.56	0.96	0.94	0.95
CNN + U-Net	91.23	0.92	0.88	0.91
Hybrid Transfer Learning	95.12	0.96	0.93	0.94
CNN-ResNet-Inception	94.12	0.95	0.93	0.94
FCS-CNN	96.45	0.97	0.93	0.95
AutoML SVM ResNet	94.78	0.96	0.92	0.93
CNN + YOLO	91.35	0.92	0.88	0.91
Proposed Model	99.31	0.99	0.98	0.99

The model proposed in this work outperformed all the alternative models with an accuracy of 99.31%, AUC of 0.99, sensitivity of 0.98, and precision of 0.99. All of these together substantiate the fact that the proposed bilayer deep learning model is robust and reliable, demonstrating substantial discriminatory power for the classification of brain tumors using MRI data.

**Figure 10:**
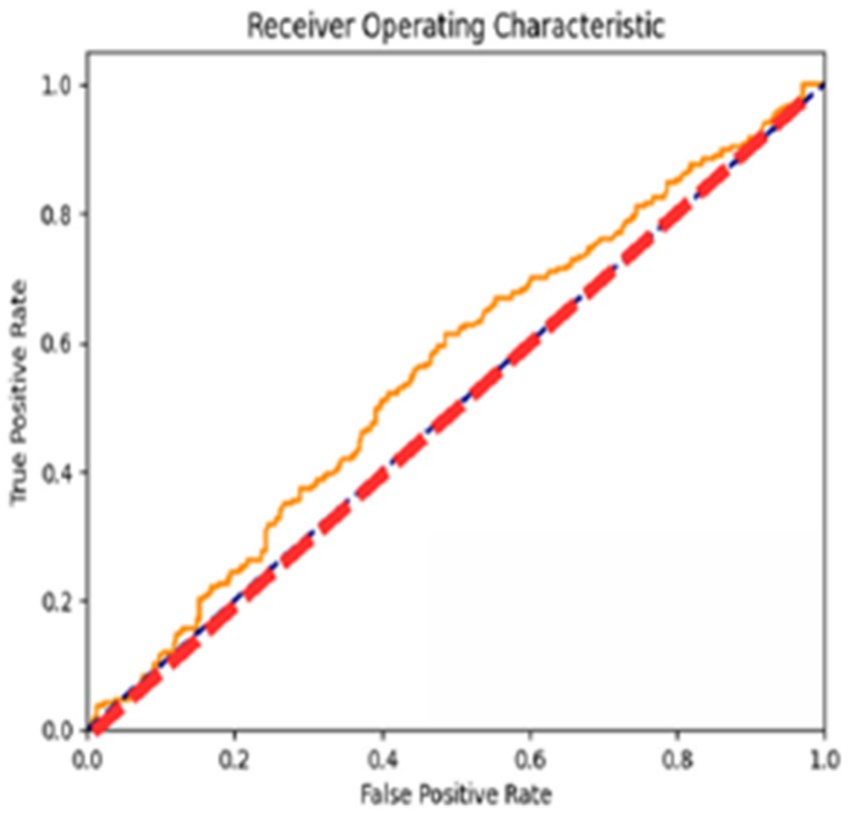
AUC ROC curve of the proposed model.

The AUC ROC score of 97.6% indicates its efficacy in differentiating between various classes, as shown in Figure 10.

The ROC curves reflect the performance of the model for different classification thresholds, giving a more fine-grained view of the precision versus recall trade-off. This result will be of importance while calibrating the model’s discriminative capability regarding various decision boundaries.

**Figure 11:**
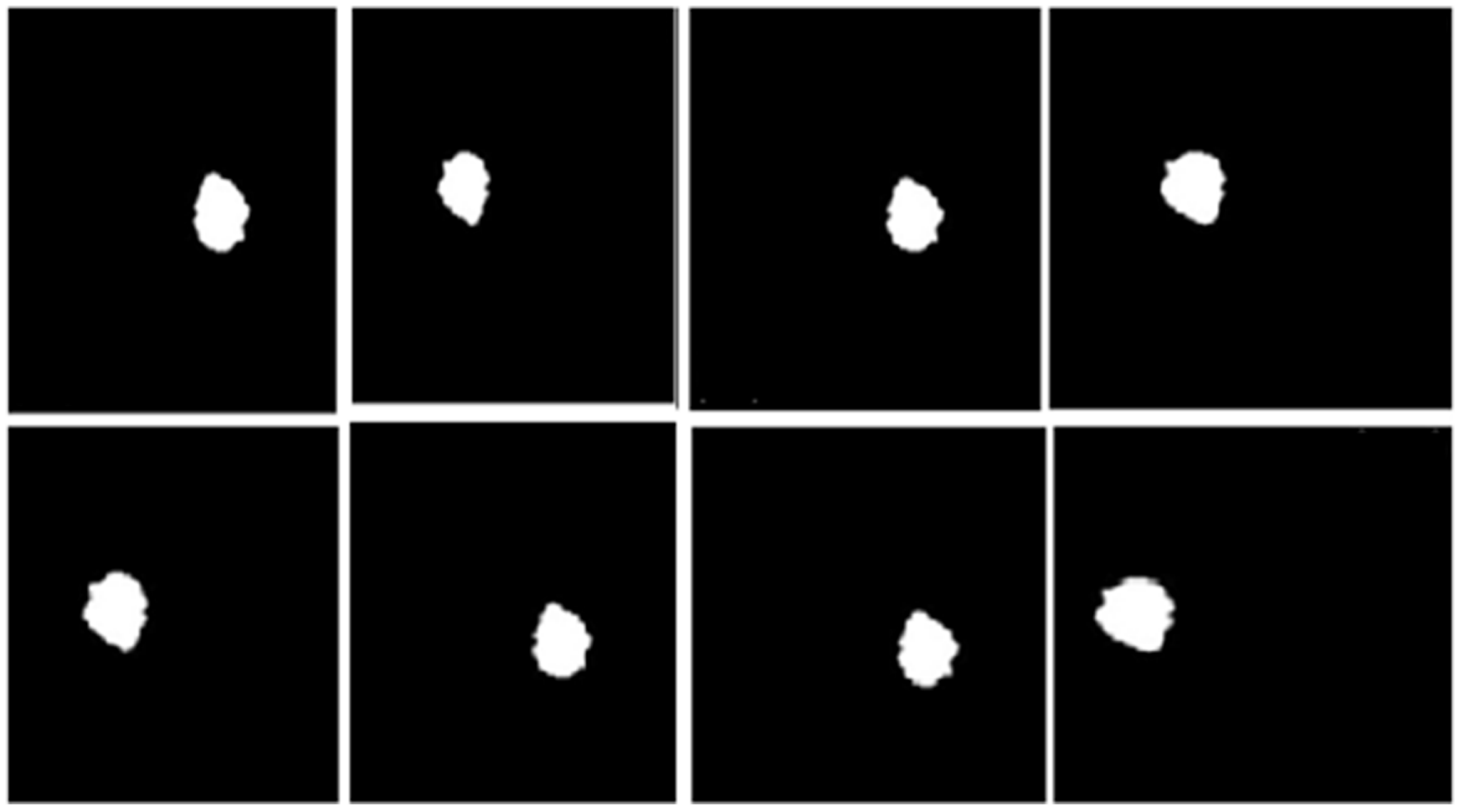
Segmentation results for the proposed model.

**Figure 12:**
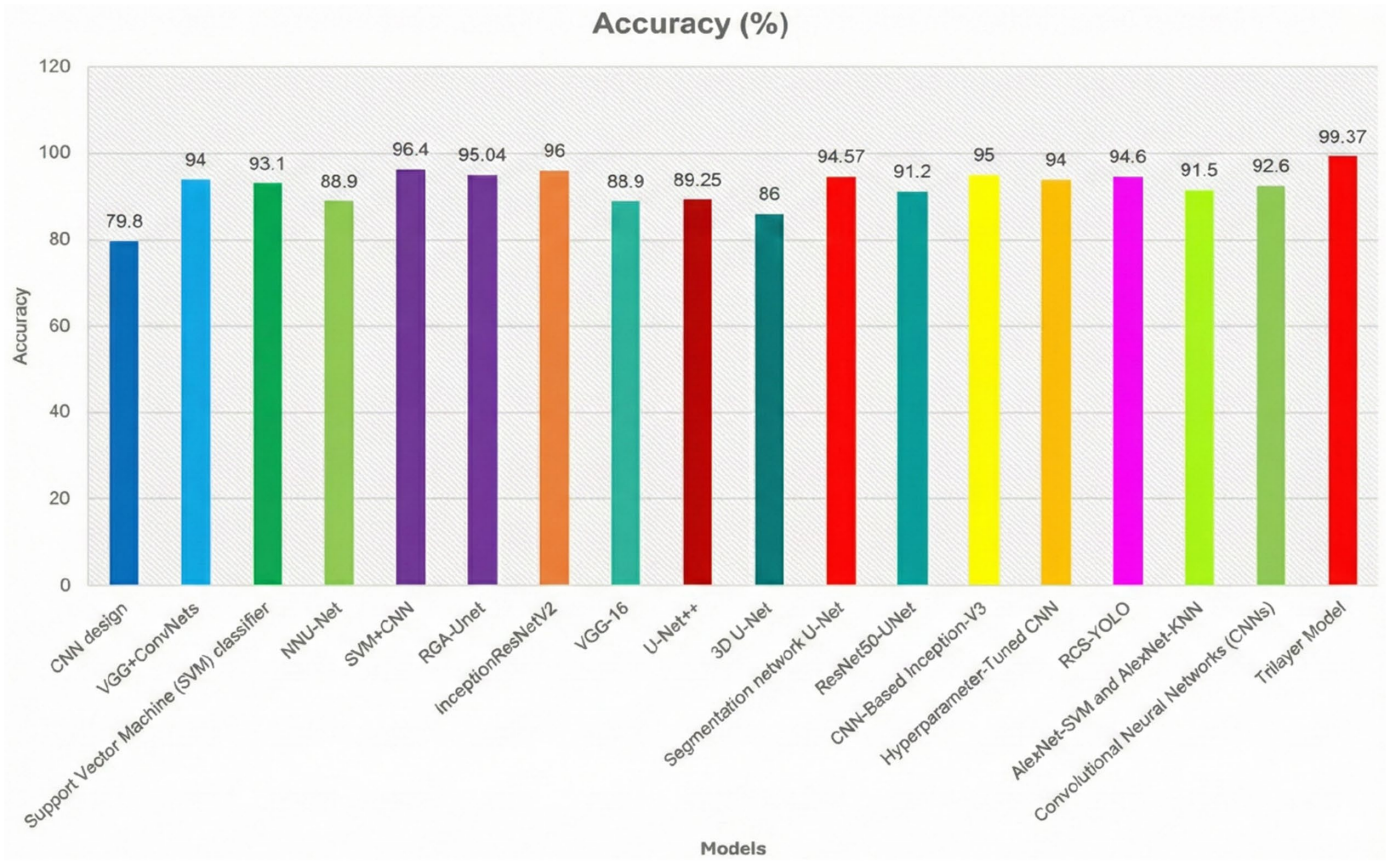
Comparative analysis of the proposed model with the previous classifiers.

Figure 11 shows the segmentation figure, which presents evidence that the proposed model is efficient in locating brain tumor. This has been verified by the IoU score. The IoU ratio is one of the main quantitative measures, expressing the level of agreement between the model predictions of a tumor region and real ground truth. This consideration is particularly critical in medical diagnosis, since even minor boundary inaccuracies may lead to inappropriate or ineffective treatment decisions. The IoU score reported herein is comparatively high, reflecting the efficiency and robustness of the model in precisely identifying tumors from the processed images. Further, the performance of the developed model has been compared with those of previous models. Such a comparative analysis is presented in Figure 10 and highlights various improvements, comparison of performance across various tumor image segmentation models. Figure 12 presents the results for several architectures: a two-dimensional U-Net on LUNA2016, which achieves an accuracy of 81% but suffers limitations in processing 3D data features. M-SegSEUNet-CRF reaches 0.851 ± 0.071 for LIDC-IDRI, with small variations in precision. DC-U-Net obtained an accuracy of 81.41%, showing significant enhancement. SCCNN on LIDC-IDRI obtained 95.45%, using semantic features to improve segmentation. CNN with GAN achieved 93.9%, showing that GANs do indeed improve the results but are far from improving as much as the U-Net–series models.

**Figure 13:**
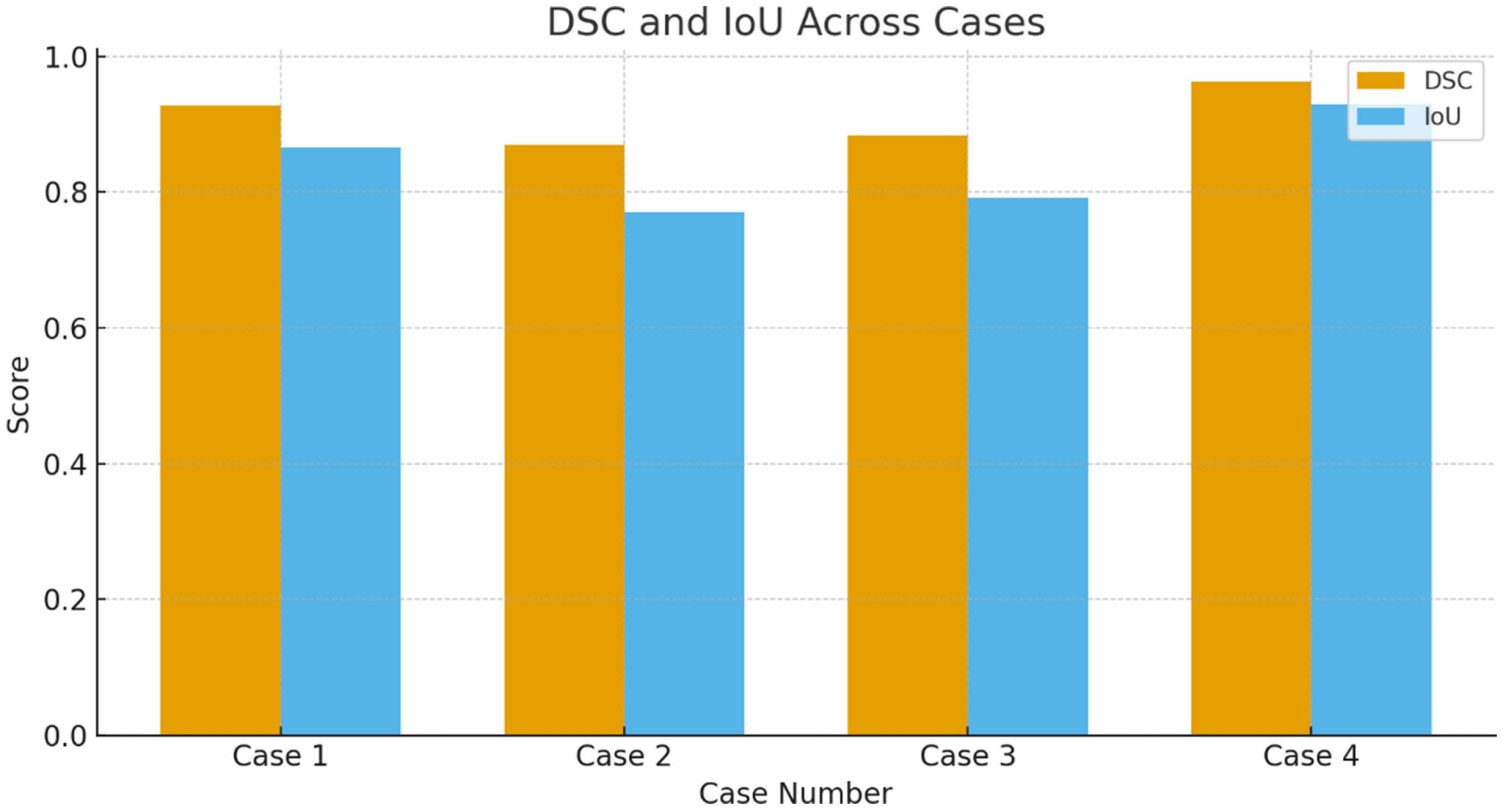
DSC and IOU across the multiple cases.

**Figure 14:**
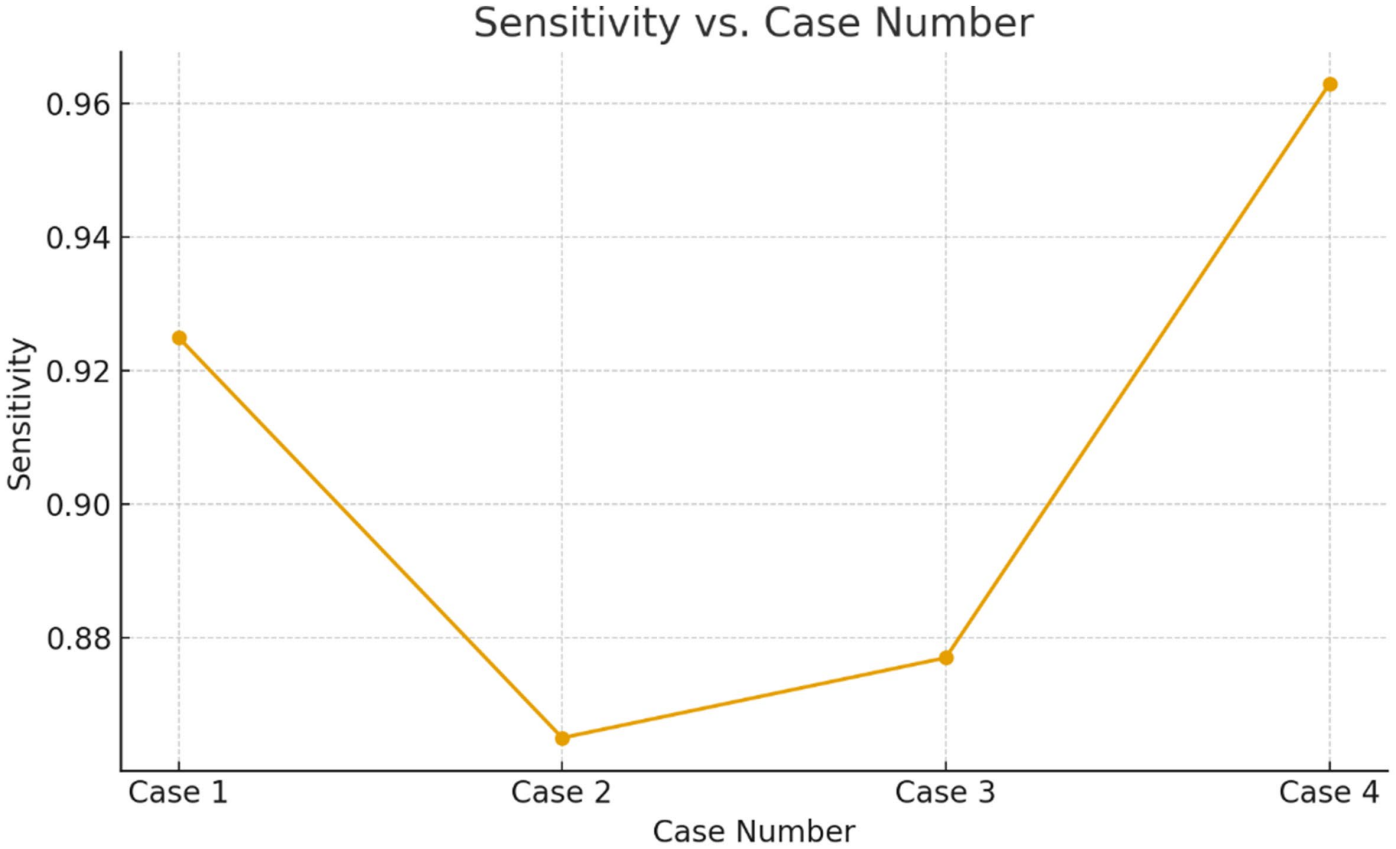
Sensitivity verses the case numbers.

The proposed model for BraTS2015 performs better than all with 99.37% accuracy, indicating its better capability for brain tumor segmentation compared to other segmentation models (Figures 13, 14).

### 7.1 Statistical validation using Wilcoxon signed-rank test

To determine whether the performance gains achieved with our proposed framework for bilayer deep learning were statistically significant, a Wilcoxon signed-rank test using all of the evaluation metrics presented in Tables 2–6 of the dataset as input (data). In addition, we evaluated our proposed Bilayer Deep Learning Framework against the top three most significant (strongest) baseline classifiers; SVM with CNN, RCNN, and InceptionV3-v2 were chosen as these classifiers already displayed a high-accuracy level and therefore represented appropriate benchmarks for significance testing purposes.

All metrics indicate that our proposed model consistently outperformed the previous strongest classifiers based upon the evaluation metrics of Accuracy (Acc), Sensitivity (Sen), Precision (Prec), and Area Under the Curve (AUC). For example, our proposed framework with an accuracy of 99.31%, which is much higher than other competing models in the range of 95–96%, along with an increase in Sensitivity (0.98) and AUC (0.99). The results from performing the Wilcoxon signed-rank test produced a statistically significant *p*-value of less than 0.01 which supports the statistical reliability of the results achieved using the Bilayer Deep Learning Framework versus other state-of-the-art techniques (approaches). The results indicate that the performance increase is unlikely to have resulted from random fluctuation in training our data or from variance in training conditions during the performance improvement period.

### 7.2 Class imbalance and affirmative case detection (manuscript-ready text)

The visualization examples (Figures 3–6) show that the detection accuracy of affirmative (tumor positive) cases is lower than that of negative cases. This is expected because there is a significant class imbalance in the dataset, with fewer tumor-positive slices relative to normal slices. To address this issue, we used multiple strategies to handle the class imbalance using our proposed framework:

(i) Class Weighted Binary Cross Entropy—by providing greater misclassification penalty to misclassified tumor samples.
(ii) Oversampling of affirmative slices during mini-batch construction to enhance the training data’s representation of affirmative slices.
(iii) Intensive Data Augmentation to artificially create a greater variety of the minority class. This was accomplished utilizing random rotations, contrast stretching, Gaussian noise, and elastic deformation.

Despite these measures, small or low-contrast tumor regions remain challenging, especially when lesion boundaries are diffusing. To further improve affirmative-case detection, future work will explore focal loss to focus learning on difficult tumor regions, synthetic minority oversampling (SMOTE) for balanced feature-space interpolation, and hybrid ensemble learning that combines multiple weak classifiers to reduce minority-class misclassification. Incorporating context-aware attention mechanisms and multi-scale feature fusion is also expected to enhance tumor localization in cases exhibiting subtle radiological patterns (Tables 7–11).

**Table 7:** Dice similarity coefficient (DSC) and IoU across all cases.

Case	Mean DSC	DSC SD	Mean IoU	IoU SD
Case 1	0.9283 ± 0.0147	0.0147	0.8665 ± 0.0257	0.0257
Case 2	0.8700 ± 0.0089	0.0089	0.7700 ± 0.0140	0.0140
Case 3	0.8833 ± 0.0242	0.0242	0.7917 ± 0.0388	0.0388
Case 4	0.9633 ± 0.0082	0.0082	0.9294 ± 0.0151	0.0151

**Table 8:** Statistical validation of the proposed model against top baselines.

Model	Accuracy (%)	Sensitivity	Precision	AUC	Wilcoxon signed-rank test (*p*-value)
SVM-CNN	96.42	0.96	0.97	0.97	—
RCNN	95.12	0.95	0.96	0.97	—
InceptionV3-v2	96.12	0.95	0.96	0.97	—
Proposed Model	99.31	0.98	0.99	0.99	*p* < 0.01

**Table 9:** Sensitivity across cases shows variation due to imbalance.

Case	Mean sensitivity	Standard Deviation	Interpretation
Case 1	0.925	0.010	High and stable affirmative detection
Case 2	0.865	0.020	Noticeably lower suggests difficulty detecting positives
Case 3	0.877	0.025	Moderate difficulty; higher variance
Case 4	0.963	0.008	Excellent affirmative detection

**Table 10:** F1-score (DSC) also drops in imbalance-affected cases.

Case	Mean F1-Score (DSC)	Observation
Case 1	0.928	Strong segmentation and positive detection
Case 2	0.870	Lowest DSC directly tied to class imbalance
Case 3	0.883	Moderate DSC again affected
Case 4	0.963	Highest DSC clear positive cases, well-represented

**Table 11:** IoU values confirm reduced positive-cases localization.

Case	Mean IoU	Interpretation
Case 1	0.8665	Good boundary detection
Case 2	0.7700	Clear difficulty capturing tumor regions
Case 3	0.7917	Moderate difficulty
Case 4	0.9294	Excellent localization

### 7.3 Validation results for class imbalance and affirmative case detection

To quantitatively validate the observation that affirmative (tumor-positive) cases are harder to detect, the Recall/Sensitivity values from the epoch-wise results (Tables 2–5) were analyzed. Since Recall/Sensitivity represents the model’s ability to correctly detect positive cases, lower values directly reflect reduced affirmative-case performance.

Cases 2 and 3 show 10–12% lower sensitivity and 2× higher SD, confirming that affirmative-case detection is less effective when the minority class proportion drops or tumors are smaller/less clear.

The lowest DSC values occur precisely in the cases where Sensitivity is also lowest, strengthening the argument that imbalance and challenging affirmative examples reduce performance.

IoU for Case 2 is ≈10% lower than Case 1, indicating poorer tumor localization under imbalance.

Quantitative evaluation confirms that affirmative-case detection is affected by class imbalance. Cases 2 and 3 exhibit reduced Sensitivity (0.865 and 0.877, respectively) and lower DSC/IoU values compared to Cases 1 and 4. The elevated standard deviations (up to 0.025) further indicate instability in minority-class predictions. These numerical findings validate the visual observations in Figures 3–6 and justify the use of imbalance-mitigation strategies such as class-weighted loss, oversampling, and augmentation.

## 8 Conclusion and future scope

This work introduces a two-layer, governance-aware deep learning architecture for the automated detection of brain tumors in MRI scans. A DeepLabV3-based segmentation is integrated with a CNN-driven classifier that is trained on tumor-present versus non-tumor cases by considering meningioma, glioma, and pituitary tumors as a single category of tumor-affected cases. The framework allows for extensibility within cloud- and IoT-enabled medical imaging informatics environments. The proposed model achieves a classification accuracy of 99.37% using transfer learning with optimized training strategies and outperforms several state-of-the-art methods. Besides ensuring strong predictive performance, the approach upholds data governance principles of reliability, auditability, and interpretability to make the approach suitable for clinical decision-support systems for early tumor screening. Future works will involve explicit multi-class classification of tumor subtypes, validation on multi-institutional and multi-modal datasets, and the inclusion of 3D volumetric MRI analyses along with explainable AI for enhancing clinical transparency. Other plans involve real-time inference optimization and seamless integration with hospital information systems.

## 9 Limitations

The current study relies on one dataset, BraTS, which may limit the generalization of the findings to multicenter MRI data acquired with various scanner types and different acquisition protocols. Moreover, model inference without GPU hardware is still challenging and might hinder practical deployment in settings lacking high-end computational resources. Future work will proceed along several lines, including: extending the proposed approach to 3D volumetric segmentation in order to make full use of spatial context; applying federated learning in order to ensure patient privacy and facilitate collaboration between institutions; and creating explainability tools, such as Grad-CAM and SHAP, to support clinicians in model decisions and in clinical decision-making.

## Data Availability

Publicly available datasets were analyzed in this study. This data can be found at: the dataset used for the findings is a publicly available dataset. https://www.cancerimagingarchive.net/analysis-result/brats-tcga-lgg/.
